# The incidence of significant venous sinus stenosis and cerebral hyperemia in childhood hydrocephalus: prognostic value with regards to differentiating active from compensated disease

**DOI:** 10.1186/s12987-020-00194-4

**Published:** 2020-04-29

**Authors:** Grant Alexander Bateman, Swee Leong Yap, Gopinath Musuwadi Subramanian, Alexander Robert Bateman

**Affiliations:** 1 Department of Medical Imaging, Newcastle Region Mail Center, John Hunter Hospital, Locked Bag 1, Newcastle, NSW 2310 Australia; 2 Newcastle University Faculty of Health, Callaghan Campus, Newcastle, NSW Australia; 3 Department of Paediatric Neurology, John Hunter Hospital, Newcastle, NSW Australia; 4 Biomedical Engineering, University of NSW, Sydney, NSW Australia

**Keywords:** Obstructive, Communicating, External, Hydrocephalus, Venous sinus stenosis, Cerebral hyperemia

## Abstract

**Background:**

Symptomatic or active hydrocephalus in children is linked to an elevation in intracranial pressure (ICP), which is likely to be multifactorial in origin. The CSF outflow resistance, venous sinus resistance and total cerebral blood flow are likely factors in the ICP elevation. The purpose of this paper is to define the incidence, site and significance of venous sinus stenosis and/or cerebral hyperemia in a cohort of children diagnosed with hydrocephalus at a tertiary referral hospital.

**Methods:**

The imaging database was reviewed over a 10 year period and the index MRI of all children between the ages of 4 months and 15 years, who were diagnosed with treatment naive hydrocephalus of any type (excluding secondary to tumor) and had magnetic resonance venography (MRV) and flow quantification were selected. Patients were compared with children undergoing an MRI with MRV and flow quantification who were subsequently shown to have no abnormality. The cross-sectional area and circumference of the sinuses were measured at 4 levels. The hydraulic and effective diameters were calculated. An area stenosis of 65% or greater was deemed significant. A total cerebral blood flow greater than two standard deviations above the mean for controls was taken to be abnormal.

**Results:**

There were a total of 55 children with hydrocephalus compared to 118 age matched control MRV’s and 35 control flow quantification studies. A high grade stenosis occurred in 56% of patients but in none of the controls (p < 0.0001). The commonest site of narrowing was in the distal sigmoid sinus. Cerebral hyperemia occurred in 13% of patients but did not occur in the controls.

**Conclusions:**

The elevation in ICP in symptomatic hydrocephalus is multifactorial. Both high grade venous stenosis and cerebral hyperemia are common in childhood hydrocephalus. High grade stenosis was noted to be a risk factor for conservative management failure. Hyperemia was a good prognostic indicator.

## Background

Hydrocephalus in children refers to a disorder of CSF physiology resulting in abnormal expansion of the CSF volume, typically it is associated with an increase in intracranial pressure [1]. In the traditional bulk flow model, CSF is secreted by the choroid plexus, passes through the ventricles, into the subarachnoid spaces to enter the venous system via the arachnoid granulations. In this model, hydrocephalus develops from an imbalance between CSF production and absorption [1]. In infants, active hydrocephalus presents with an increasing head circumference, irritability, vomiting, bulging of the anterior fontanel or splaying of the cranial sutures. Beyond infancy, active hydrocephalus typically presents with a combination of headache, vomiting, loss of developmental milestones, diplopia or papilledema [1]. Compensated hydrocephalus is associated with enlargement of the CSF spaces but a lack of symptoms, no change in ventricular size over time and a head circumference which is not crossing centile lines [2]. Compensated hydrocephalus may come at the risk of subclinical changes to intellectual performance and possible decompensation at a later date mandating continuing surveillance [2]. It can be hypothesized that in active hydrocephalus the symptoms may be related to an elevation in intracranial pressure (ICP) and in compensated hydrocephalus the ICP is likely normal. The ICP depends on several factors and is modeled using Davson’s equation:

1 $$ICP = FR_{CSF} \times R_{out} + SSS_{p}$$

where *FR*_*csf*_ is the CSF formation rate, *R*_*out*_ is the CSF outflow resistance and *SSS*_*p*_ is the superior sagittal sinus pressure. The sagittal sinus pressure itself depends on Ohm’s law, i.e. the product of the outflow resistance and blood flow through the outflow plus the jugular bulb pressure [3]. So Eq. (1) can be expanded to:

2 $$ICP = FR_{CSF} \times R_{out} + TCBF \times R_{ven} + CVP$$

where *TCBF* is the total blood flow leaving the capillaries to enter the venous system, *R*_*ven*_ is the venous outflow resistance from the sagittal sinus to the jugular bulbs and *CVP* is the central venous pressure. Each of these 5 variables has been shown to independently be associated with active hydrocephalus in children in the literature. CSF formation rates have been noted to be increased by 2–5 times in the hydrocephalus secondary to choroid plexus papilloma [4]. The CSF outflow resistance is increased in hydrocephalus secondary to acute hemorrhage and meningitis [5]. A Vein of Galen aneurysm produces a high flow arteriovenous shunt and elevated sinus pressure. In 9 of 21 cases of hydrocephalus secondary to this condition, the venous pressure was judged to be the sole cause of the ventricular enlargement [6]. In achondroplasia, fixed outflow stenosis at the skull base causes hydrocephalus [7]. Hydrocephalus has been documented to occur in children with raised central venous pressure secondary to cardiac anomalies and jugular vein thrombosis [8]. Thus, there is strong evidence an elevation in ICP is behind the symptoms of active childhood hydrocephalus. Indeed, Rosman and Shands suggested, elevated venous pressure causes idiopathic intracranial pressure (IIH) in adults but causes hydrocephalus in children, the difference in outcome appears to depend on whether the cranial sutures are patent or closed [8].

An increase in CSF formation rate and an elevation in central venous pressure are likely to be rare associations of hydrocephalus but increased R_out_, venous sinus stenosis and an increase in total cerebral blood flow (hyperemia) are possibly more common. In 1962 Kinal described bilateral distal high grade venous outflow stenoses in 4 infants with hydrocephalus using venography following direct sinus injection through the anterior fontanel [9]. In 1965, Shulman and Ransohoff described 15 children with hydrocephalus in whom there was an elevation in SSS pressure due to sinus narrowing with loss of the driving force across the arachnoid villi [10]. Finally, these stenoses have been shown to be hemodynamically significant [11]. With regards to cerebral hyperemia, a number of cases have been documented in the literature [3, 12]. What is not currently known is, (1) what is the incidence of each of these variables in a population of hydrocephalic children? and (2) what is the relative significance/prognostic value of each ? Thus, the purpose of the current study is to define the incidence, site and significance of venous sinus stenosis and/or cerebral hyperemia in a cohort of children referred for MRI imaging of hydrocephalus at a tertiary referral hospital over a 10 year period.

## Methods

### Subjects

The radiology information system at a tertiary referral hospital was retrospectively interrogated to retrieve the data from children between birth and 15 years of age who had an MRI with MR venography and flow quantification, for the investigation of any form of hydrocephalus between January 2009 and January 2019. MRV and flow quantification has been a standard component of a comprehensive MRI examination of childhood hydrocephalus over this entire period. Hydrocephalus was defined on imaging as a ventricular size index (width of the ventricles in the frontal region divided by the width of the frontal lobes) greater than 0.3 with a disproportionate reduction in subarachnoid space width or size of sulci (to exclude atrophy) for communicating/obstructive hydrocephalus. In many instances an initiating cause of the hydrocephalus such as hemorrhage or infection was evident or there was progressive enlargement of the ventricles on subsequent imaging. If there was a question whether the ventriculomegally was due to ex-vacuo dilatation, an increase in the temporal horns of the ventricles was taken to suggest hydrocephalus [13]. In external hydrocephalus, the definition of an increased frontal subarachnoid space above 10 mm in thickness and head circumference above the 97th percentile or rapid crossing of centile lines was used [14]. Obstructed patients had no flow through the aqueduct on flow quantification. Patients were judged to have active hydrocephalus if symptomatic of raised ICP, and or have a rapidly enlarging head and or enlarging ventricles. Any evidence of transependymal CSF spread was documented by reviewing the T2 images. Compensated hydrocephalus was diagnosed if these latter findings were lacking. Initially there were 93 patients with hydrocephalus returned. Of these, 3 were excluded due to technically inadequate imaging. 12 patients were excluded because there were no preoperative studies available. There were 17 patients in whom obstructive hydrocephalus was due to a posterior fossa tumor. Using the current methodology 11/17 or 65% had a significant outflow stenosis and 2/17 or 12% a non-significant stenosis. Twelve of these 17 patients (who had venous sinus stenosis) were the subject of a prior publication [15] and these were excluded to limit literature duplication. The clinical findings and follow-up for these patients can be found in this publication [15]. There were 6 infants aged between 7 and 48 days with hydrocephalus (all of whom had high grade venous sinus stenosis) but they could not be included due to the lack of available controls in such a young cohort. There remained 55 patients, 35 male and 20 female. There were three types of hydrocephalus noted i.e. external, obstructed and communicating. Twenty-two patients were judged to have active hydrocephalus and 33 compensated. One hundred and eighteen control patients were enrolled. They had an MRI study for indications not related to headaches, large head, raised intracranial pressure or hydrocephalus, in which the subsequent MRI was found to be entirely normal. Twenty-nine percent were imaged for investigation of epilepsy, 24% for presumed transient ischemic event, 18% to rule out intracranial extension of a skin lesion and 6% for cerebral palsy. All 118 controls had an MRV and 35 also had flow quantification. There were 65 males and 53 females with MRV and 16 female and 19 males with flow quantification. The patients and controls were split into 3 age groupings (see Table 1). The age groupings were selected to cover the rapid increase in sinus size and blood flow in the early ages which later stabilized in the older ages. The clinical findings for the patients are listed in Table 2. As is common in MRI of children, those aged between 4 months and 7 years underwent a general anesthetic with both pulse oximetry and capnography monitoring. Conscious sedation is not utilized for children in our unit. The partial pressure of carbon dioxide was maintained between 35 and 40 mmHg by the anesthetist. There were no differences between the treatment of the controls and hydrocephalus patients.

**Table 1:** Sinus size and blood flow by age

	Age years	SSS Hd mm	TS Ed mm	SS Ed mm	DSS Ed mm	TCBF ml/min
		Control	< 1 year old			
Mean	0.67	6.1	7.7	6.1	6.7	640
SD	0.31	0.8	0.8	1.1	1.0	150
		Hydrocephalus	< 1 year old			
Mean	0.64	5.1	7.1	5.1	3.6	640
SD	0.19	0.9	1.1	1.4	1.7	190
ttest	0.74	0.008^a^	0.24	0.08	< 0.0001^a^	0.97
		Control	1 to 4 years old			
Mean	2.00	7.0	8.8	8.0	7.3	1080
SD	0.46	0.7	0.9	1.3	1.6	210
		Hydrocephalus	1 to 4 years old			
Mean	1.63	6.5	8.2	6.4	5.0	1160
SD	0.53	0.9	1.4	2.0	2.4	310
ttest	0.07	0.03	0.10	0.002^a^	0.0004^a^	0.55
		Control	4 to 15 years old			
Mean	10.42	6.7	8.1	8.3	8.1	990
SD	3.42	0.8	1.2	1.4	1.5	210
		Hydrocephalus	4 to 15 years old			
Mean	10.35	5.4	6.8	8.5	6.2	1070
SD	3.24	1.6	2.2	2.0	2.9	320
ttest	0.94	< 0.0001^a^	0.001^a^	0.53	0.0005^a^	0.40

*DSS* distal sigmoid sinus, *Ed* effective diameter, *Hd* hydraulic diameter, *ml/min* milliliters per minute, *mm* millimeters, *SD* standard deviation, *SSS* superior sagittal sinus, *SS* sigmoid sinus, *TCBF* total cerebral blood flow, *TS* transverse sinus

^a^Significance < 0.0125

**Table 2:** Sinus size and blood flow by hydrocephalus type

Case	Age years	Clinical	SAS/VSI mm	TCBF ml/min	SSS Hd mm	TS Ed mm	SS Ed mm	DSS Ed mm	Max stenosis %	Stenosis site
External hydrocephalus										
1	0. 41	Seizures SDH active VPS	10	586	4.00	4.86	4.98	4.31	60	TS
2	0.46	Macrocephaly floppy active	11	471	4.28	7.52	7.06	4.64	–	–
3	0.59	Macrocephaly compensated	10	577	6.41	6.47	3.13	3.07	*79*	*DSS*
4	0.72	Macrocephaly compensated	10	707	5.70	7.76	6.36	2.49	*86*	*DSS*
5	0.90	Premature compensated	15	417	5.06	3.69	4.55	4.06	*77*	*TS*
6	0.95	Premature IVH compensated	12	785	5.37	6.96	6.60	4.17	61	DSS
7	0.96	Macrocephaly vomiting active	14	695	5.89	6.51	6.39	6.12	–	–
8	1.14	Aural atresia compensated	11	717	5.91	8.26	6.60	3.67	*77*	*DSS*
9	1.46	Macrocephaly compensated	10	1329	7.53	6.73	8.20	6.79	–	–
Mean	0.84			698	5.57	6.53	5.98	4.37		
SD	0.34			266	1.07	1.44	1.51	1.36		
Obstructed hydrocephalus										
10	0.34	Optic nerve hypoplasia compensated	0.41	382	3.37	5.27	5.17	3.33	*75*	*DSS*
11	0.36	Macrocephaly active TECSF VPS	0.71	362	3.93	6.27	3.89	3.01	*80*	*DSS*
12	0.52	Macrocephaly active ETV	0.50	541	4.72	7.06	6.06	2.20	*89*	*DSS*
13	4.43	Premature compensated	0.46	692	6.89	9.89	8.97	9.51	–	–
14	6.00	Congenital compensated	0.49	1598^a^	7.73	8.94	6.70	8.15	–	–
15	7.84	Headaches blurred vision TECSF active ETV	0.53	706	3.60	5.57	4.46	3.66	*79*	*DSS*
16	7.50	Papilledema TECSF active ETV	0.38	892	4.79	4.90	8.33	0.00	*95*	*JB*
17	9.98	Noonan syndrome compensated	0.76	1241	5.67	4.62	5.50	6.00	*67*	*TS*
18	12.77	Dyspraxia TECSF active ETV	0.56	1375	5.34	6.00	4.32	4.32	*73*	*SS*
19	14.74	Papilledema TECSF active ETV	0.40	832	4.93	3.59	6.16	8.69	*80*	*TS*
Mean	6.45			862	5.10	6.21	5.96	4.89		
SD	5.14			420	1.39	1.95	1.68	3.10		
Communicating hydrocephalus										
20	0.33	Achondroplasia compensated	0.46	337	5.11	8.20	5.38	0.00	*95*	*JB*
21	0.47	Increasing HC active	0.48	745	3.76	9.29	2.94	7.39	*76*	*SS*
22	0.51	Meningitis compensated	0.40	818	4.70	7.76	6.15	4.17	61	DSS
23	0.52	Premature TECSF active VPS	0.75	693	5.74	5.94	2.91	1.78	*93*	*DSS*
24	0.64	Papilledema active	0.32	645	4.87	10.31	5.00	2.73	*83*	*DSS*
25	0.64	Macrocephaly compensated	0.47	688	6.37	6.98	5.61	4.97	–	–
26	0.66	Craniofacial cutaneous compensated	0.35	518	4.89	7.85	5.69	5.89	–	–
27	0.66	Meningitis compensated	0.39	706	7.51	6.60	4.13	3.59	*71*	*DSS*
28	0.77	Increasing HC active	0.47	627	5.16	7.26	2.67	1.49	*95*	*DSS*
29	0.82	Increasing HC TECSF active	0.33	1210^b^	4.73	7.83	4.59	3.80	*68*	*DSS*
30	0.89	Premature active VPS	0.46	902	4.81	6.98	4.15	3.32	*75*	*DSS*
31	0.91	Increasing ventricles TECSF active VPS	0.44	748	4.92	6.04	3.55	2.62	*85*	*DSS*
32	1.07	Macrocephaly compensated	0.39	787	6.07	8.27	5.25	4.53	64	DSS
33	1.12	Macrocephaly compensated	0.43	1054	6.45	8.46	3.60	3.89	*80*	*SS*
34	1.20	Macrocephaly active VPS	0.46	1141	6.39	8.32	3.35	2.08	*92*	*DSS*
35	1.32	Achondroplasia increasing HC active	0.41	1103	6.74	6.89	4.67	0.00	*95*	*JB*
36	1.36	Macrocephaly increasing HC active ETV	0.50	853	5.98	6.56	7.62	7.35	–	–
37	1.36	Macrocephaly compensated	0.39	980	6.76	8.98	4.64	6.73	*67*	*SS*
38	1.48	Premature increasing HC IVH active	0.40	1467	5.46	5.41	5.24	4.35	*67*	*DSS*
39	1.63	Macrocephaly compensated	0.42	1180	7.49	9.42	7.94	4.91	58	DSS
40	1.84	Congenital compensated	0.42	816	6.33	7.64	6.68	6.31	–	–
41	1.89	Macrocephaly compensated	0.42	1308	4.96	9.63	5.15	2.17	*92*	*DSS*
42	2.35	Macrocephaly compensated	0.40	1754^a^	6.77	10.89	9.86	6.85	–	–
43	2.40	Premature compensated	0.39	1632^a^	5.86	8.62	8.12	5.89	–	–
44	2.84	Macrocephaly compensated	0.42	1279	8.49	9.03	8.76	9.36	–	–
45	5.36	Behavioral issues compensated	0.38	1474^a^	7.95	7.52	9.25	6.54	–	–
46	5.44	Talipes compensated	0.46	692	7.71	6.77	7.03	7.22	–	–
47	6.60	Meningitis compensated	0.36	755	2.61	5.28	8.29	7.48	*85*	*SSS*
48	8.52	Premature IVH compensated	0.42	1001	5.37	12.77	11.42	7.87	–	–
49	9.69	Achondroplasia increasing HC active	0.45	786	5.05	7.99	9.15	0.00	*95*	*JB*
50	11.10	Dysmorphic compensated	0.54	1044	5.37	6.61	9.45	6.52	–	–
51	11.10	Seizures compensated	0.57	1435^a^	4.97	8.28	10.69	8.60	–	–
52	11.15	Achondroplasia compensated	0.42	1727^a^	7.13	4.78	8.13	6.89	*65*	*TS*
53	12.42	Achondroplasia vomiting active	0.56	1030	2.86	5.16	6.67	0.00	*95*	*JB*
54	14.07	Crouzon’s compensated	0.43	821	5.08	7.27	10.42	5.72	50	DSS
55	14.45	Macrocephaly compensated	0.48	1126	6.45	8.02	9.86	9.28	–	–
Mean	3.88		0.44	997	5.75	7.77	6.50	4.78		
SD	4.49		0.08	344	1.31	1.66	2.50	2.71		

Italic values indicate significant stenosis

*DSS* distal sigmoid sinus, *Ed* effective diameter, *ETV* endoscopic third ventriculostomy, *HC* head circumference, *Hd* hydraulic diameter, *IVH* intraventricular hemorrhage, *JG* jugular bulb, *ml/min* milliliters per minute, *mm* millimeters, *SAS* subarachnoid space thickness, *SD* standard deviation, *SDH* subdural hematoma, *SSS* superior sagittal sinus, *SS* sigmoid sinus, *TCBF* total cerebral blood flow, *TECSF* transependymal CSF flow, *TS* transverse sinus, *VPS* ventriculoperitoneal shunt, *VSI* ventricular size index

^a^2 SD above mean, ^b^3 SD above mean

### MR and analysis

**Fig. 1:**
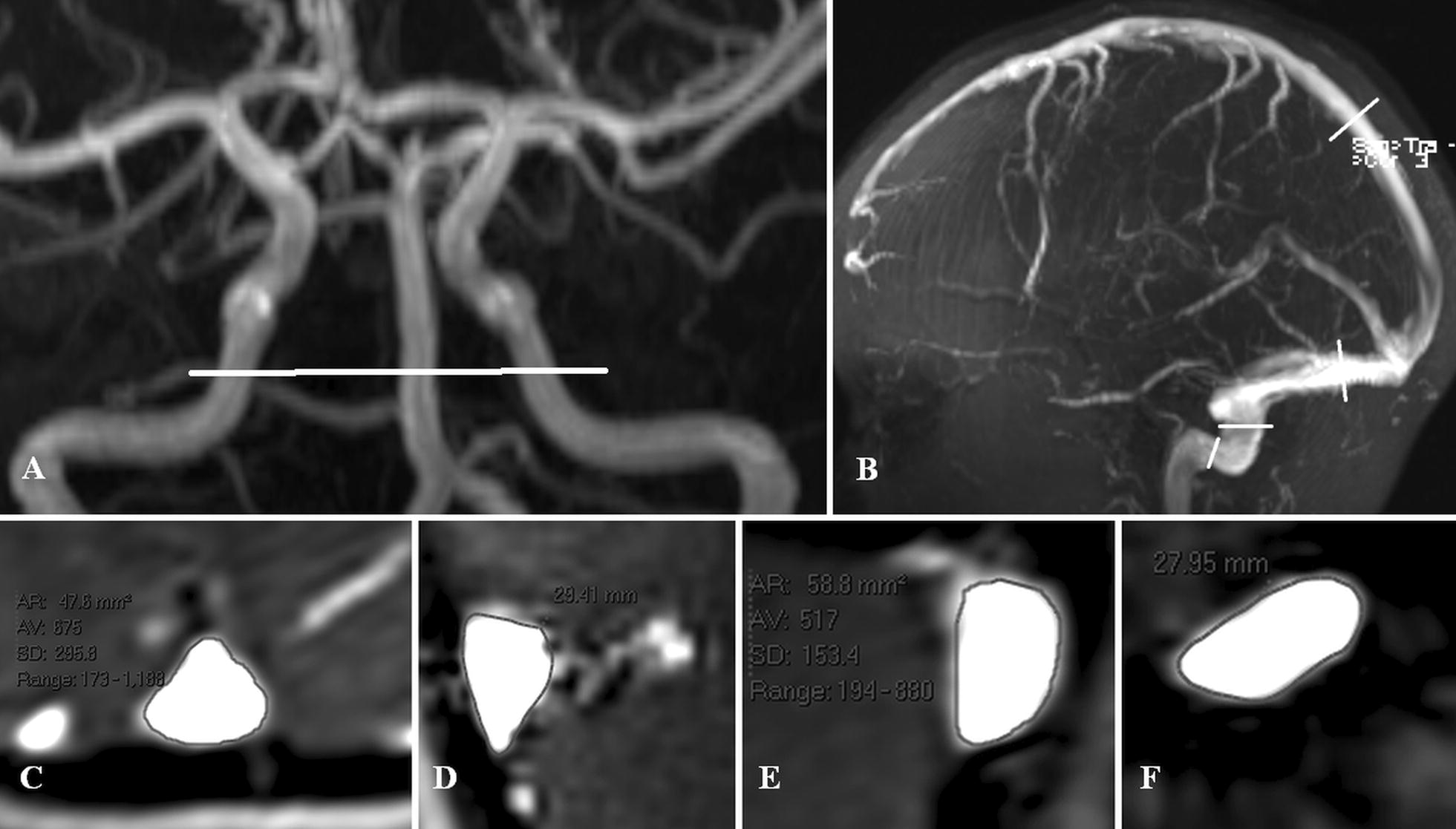
Site of flow acquisition and venous sinus measurement in a control patient. **a** An MRA of a 4.7 year old male with seizures who had a normal MRI. The while line indicates the level of the flow quantification acquisition. The acquisition crosses both carotid and basilar arteries at the skull base. **b** An MRV of the same child with while lines showing the sites of the cross-sectional measurements. From superior to inferior these are the sagittal sinus, transverse sinuses, vertical portion of the sigmoid sinuses and the horizontal portion of the sigmoid sinuses. **c** The cross-sectional image of the sagittal sinus which is triangular. **d** The cross-sectional image of the right transverse sinus which is triangular. **e** The cross-sectional image of the left vertical sigmoid sinus which is oval. **f** The cross-sectional image of the horizontal portion of the sigmoid sinus which is oval

All patients were imaged on a 3.0 T superconducting magnet (Avanto; Seimens, Erlangen Germany). In all patients, a standard brain MRI consisting of 3DT1 sagittal, T2 axial, FLAIR axial and diffusion weighted axial images was performed. An MR phase contrast flow quantification sequence was acquired with retrospective cardiac gating. The TR was 26.5 ms, TE 6.9 ms, flip angle 15º, slice thickness 5 mm, matrix 192 × 512, FOV 150 and a single excitation. The velocity encoding value was 150 cm/sec with the plane set to pass through the skull base to cross the carotid and basilar arteries (see Fig. 1a). A time of flight MRV acquisition was performed in the off sagittal plane. The MRI imaging was sourced from the hospital picture archiving and communication system (PACS) and therefore all measurements were performed on the original data.

Areas of interest were placed around the carotid and basilar arteries for all patients, to give the total arterial inflow at the skull base by summing the individual flows. Background subtraction was used to remove the effect of eddy currents. The MRV data was reformatted to display the cross-section of the sinuses at the midpoints of the distal half of the sagittal sinus, the transverse sinuses, the vertical segment of the sigmoid sinuses and the narrowest section of either the horizontal portion of the sigmoid sinuses or the proximal jugular veins (see Fig. 1b). If an accessory occipital sinus was found this was also measured. At each site the cross-sectional area of the sinus and the wetted circumference was measured using the scanners measurement tool (see Fig. 1c–f). The hydraulic diameter of each cross-section was calculated using the formula:

3 $$Hd = {\raise0.7ex\hbox{${4A}$} \!\mathord{\left/ {\vphantom {{4A} {Circ}}}\right.\kern-0pt} \!\lower0.7ex\hbox{${Circ}$}}$$

where *Hd* is the hydraulic diameter, *A* is the cross-sectional area of the sinus and *Circ* is the wetted circumference of the sinus. In the areas below the Torcular, there was more than one parallel pathway for blood to flow, the effective diameter of each segment was calculated using the formula:

4 $$D_{e} = \left( {\varSigma D_{i}^{{{\raise0.7ex\hbox{$5$} \!\mathord{\left/ {\vphantom {5 n}}\right.\kern-0pt} \!\lower0.7ex\hbox{$n$}}}} } \right)^{{{\raise0.7ex\hbox{$n$} \!\mathord{\left/ {\vphantom {n 5}}\right.\kern-0pt} \!\lower0.7ex\hbox{$5$}}}}$$

where *D*_*e*_ is the effective diameter, *D*_*i*_ is the hydraulic diameter of each parallel sinus segment and *n* is the number of parallel segments. In the cerebral circulation a hemodynamically significant stenosis is usually taken to be between 60 and 70% [16]. A search was made in both the control and patient groups for non-significant or significant stenoses. The non-significant stenoses were defined as any segment with an area calculated utilising the effective diameter, which was reduced by 50–65% compared to the mean for each control age group. Stenoses were deemed significant if they were greater than 65% by area.

Mean and standard deviations were obtained for each group. A Shapiro–Wilk Test was used to test for normality of the data. Differences between groups were tested using a non-paired t-test with a confidence level of 0.05 set for the blood flow measurement and a level of 0.0125 for the stenosis measurements following Bonferroni correction to reduce the possibility of family-wise error.

## Results

The blood flow and sinus size findings arranged by age are summarized in Table 1 and arranged by hydrocephalus type in Table 2.

In the control groups, there was a rapid maturation of all of the sinuses between the 4 month -1 year and the 1–4 years age groups, with the sinuses being on average 0.9 mm, 1.1 mm and 1.9 mm larger for the sagittal sinus, transverse sinus and the vertical portion of the sigmoid sinus respectively (p = 0.004, 0.002 and 0.0004). Following 4 years of age, the sinuses had largely attained full size with no significant difference in the second and third controls groups for any of the sinuses. In no control patient was the jugular bulb smaller than the distal sigmoid sinus. With regards to focal stenoses, the average area for each segment in each age control group was calculated using the effective diameter and this was compared to each individual in the control group. There were no non-significant stenoses in the youngest grouping, one 50% stenosis in the proximal sigmoid in the second group and 4 stenoses between 57% and 63% in the third group (1 transverse sinus, 1 proximal sigmoid and 2 distal sigmoid) for a total of 5/118 or 4.2%. There were no significant stenoses greater than 65% in the controls.

The blood flow was also noted to peak early, being 69% higher for the 1–4 years age group compared to the 4 months–1 years age group (p = 0.006) but there was no significant difference between the older two control groups.

In the 4 month–1 year hydrocephalus patients, the sagittal sinus was 1.0 mm smaller on average and the distal sigmoid sinus 3.1 mm smaller than the controls (p = 0.008 and < 0.0001 respectively), giving an average sinus area reduction of 33% and 71% respectively. For the 1–4 years age group, the distal portion of the sigmoid sinus was 2.3 mm smaller than age matched controls (p = 0.0004), giving an average area stenosis of 53%. In the 4–15 age group, the sagittal sinus was 1.3 mm smaller, transverse sinus 1.3 mm smaller and the distal sigmoid 1.9 mm smaller on average than controls (p < 0.0001, 0.001 and 0.0005 respectively), giving 35%, 30% and 41% average stenosis for these segments. With regards to individual focal stenoses, there were 6/55 or 11% of the hydrocephalus patients with stenoses between 50 and 65% and 31/55 or 56% of hydrocephalus patients with significant stenosis (see Table 2 for details). There was no significant difference between controls and patients regarding average blood flow, but 7/55 or 13% of patients had blood flows greater than 2 standard deviations above the mean and 2/55 or 4% greater than 3 standard deviations above the mean compared to age matched controls (see Table 2 for details). Two of the seven hyperemic patients had a significant venous stenosis but 5 of the seven had no evidence of stenosis at all.

### Follow up

In twelve of the active hydrocephalus patients treatment was instigated with 6 undergoing third ventriculostomy and 6 ventriculoperitoneal shunt. In 8 of the 12 treated patients there was a follow up MRI examination. In six patients, the previously noted stenosis had resolved completely and the patients were all noted to be asymptomatic. There were two shunt patients (numbers 11 and 34) and four third ventriculostomy patients (numbers 12, 18, 19 and 38) with this good result. There were two patients with a poor clinical result, Patient 15 who had obstructed hydrocephalus underwent a third ventriculostomy and continued to have early morning headaches and also developed interval papilledema. Pre-op this patient had tandem lesions with a 71% stenosis of the sagittal sinus and 79% stenosis of the distal sigmoid sinus. Following the third ventriculostomy, despite the stoma being patent, the sagittal sinus residual stenosis was 59% and the residual distal sigmoid sinus stenosis was 46%. Patient 16 had obstructed hydrocephalus and underwent third ventriculostomy. Despite the ventriculostomy being patent, the 79% stenosis of the jugular bulb was unchanged and she complained of blurred vision and daily headaches. A ventriculoperitoneal shunt was placed and the stenosis again failed to change. She continued to complain of daily headaches.

In two of the hyperemic patients there was follow up to gauge the changes in blood flow with time. Patient 38 from Table 2 had an index TCBF just below 2 SDs above the mean, but by his 7th year, the value had reached 1560 ml/min. Two years later the flow was within the normal range at 1030 mls/min. Similarly, patient 45 had an index TCBF of 1474 mls/min, which increased to 1763 in the 7th year of life but came back into the normal range at 10 years old at 1020 ml/min.

## Discussion

This study compares the hydraulic effectiveness of varying segments of the venous outflow. Unfortunately, the differing segments have widely differing shapes in cross-section. As seen in Fig. 1c–f, the sagittal and transverse sinuses tend to be triangular in cross-section, whilst the sigmoid sinuses tend to be oval. The flow through a triangular or oval venous sinus is much less efficient than a cylindrical one [17]. The hydraulic diameter Eq. (3) provides the diameter of a cylindrical sinus which is equivalent in hydraulic effect to the corresponding triangular or oval sinus [18]. This makes direct comparison between sinuses more accurate. In addition to the shape, the sinuses below the Torcular have parallel pathways. The normal anatomy could include one dominant right or left pathway only, or between 2 and 3 variously sized pathways. In order to be able to compare each of these variations directly, Eq. (4) (which is based on the Darcy-Weisbach equation) was used. The diameter of multiple tubes in parallel can be reduced to an equivalent diameter of a single tube by using this equation [19]. It can be seen that despite the great variation in individual anatomy which occurs beyond the torcular, the average standard deviation for the effective diameters of the controls was 15% of the mean indicating despite all of the variations, each were providing a similar outflow resistance.

In an infant below 1 year of age, a normal ICP averages 3.7 mmHg [20]. In 16 infants with active hydrocephalus, the mean ICP was 11.7 mmHg [21]. Suggesting a pressure increase of 8 mmHg is significant at this age. Similarly, in children between 1 and 18 years old, the normal average CSF opening pressure is 14.6 mmHg [22]. In the revised criteria for symptomatic IIH in children, an elevated opening pressure above 20.6 mmHg is required [23]. From this we can deduce an elevation of 6 mmHg is required for a child to be symptomatic from a raised ICP. This later finding fits in well with those of Blomquist et al. who noted, in 6 children with active hydrocephalus, the ICPs were 7 mmHg higher than 9 children with spontaneously arrested hydrocephalus (analogous to compensated hydrocephalus) who were asymptomatic [5]. Therefore, a 6–8 mmHg rise in ICP is required for a child to have symptomatic active hydrocephalus.

### Venous stenosis

**Fig. 2:**
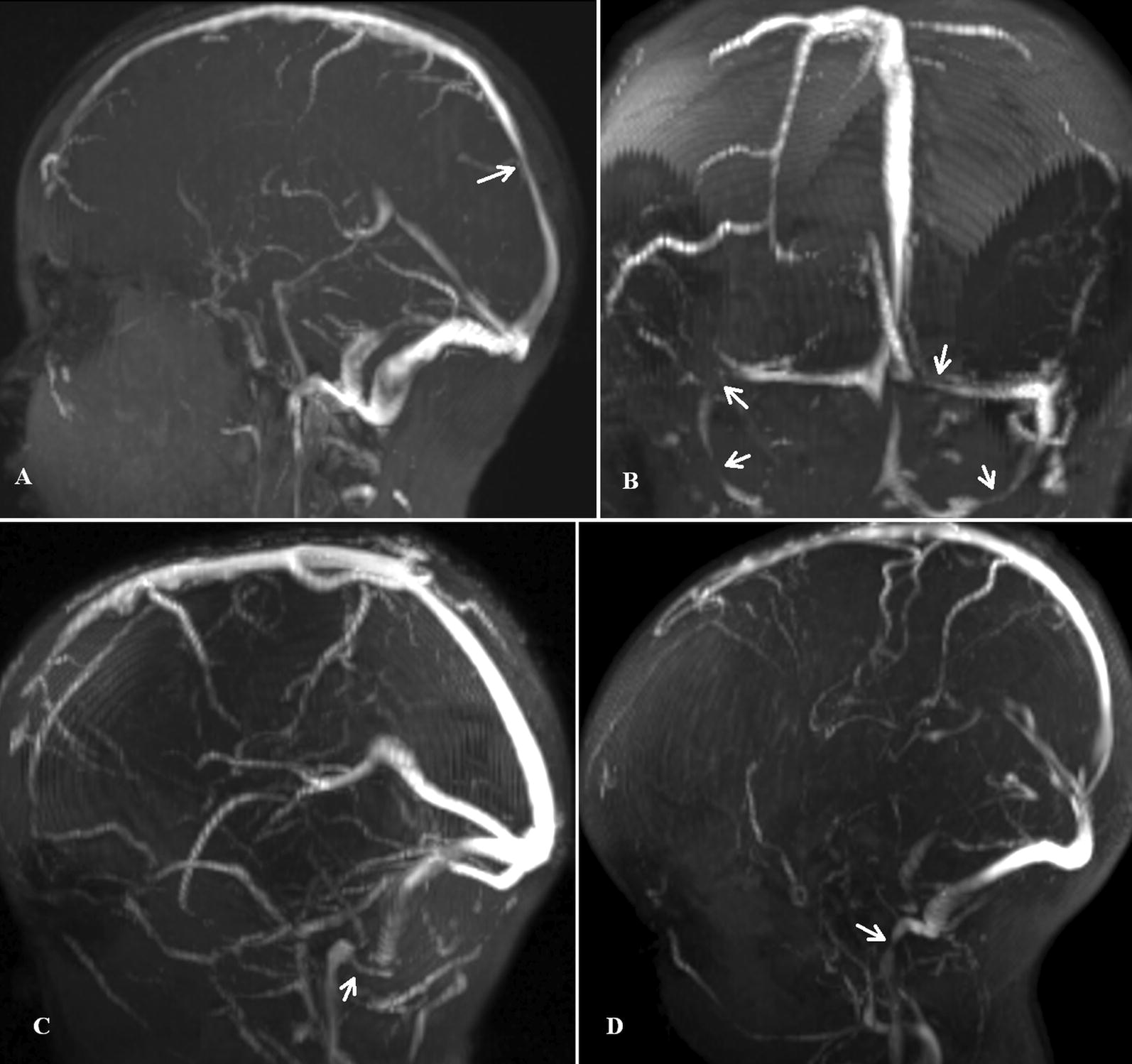
Site of venous stenosis in four patients with hydrocephalus. **a** The MRV of case 47, a 6.6 year old female with communicating hydrocephalus secondary to meningitis who has an 85% stenosis of the sagittal sinus (arrow). **b** The MRV of case 5, a 0.9 year old male with external hydrocephalus who has high grade stenoses of the both transverse and sigmoid sinuses (arrows). Note the accessory occipital sinus in the midline is also stenosed at its origin. **c** The MRV of case 28, a 0.8 year old female with communicating hydrocephalus who has a 95% stenosis of the distal sigmoid sinus (arrow). **d** The MRV of case 20, a 0.33 year old male with communicating hydrocephalus secondary to achondroplasia who has a 95% stenosis of the jugular bulb (arrow). Note in this instance the jugular bulb was smaller than the adjacent sigmoid sinus so it was measured instead

In their review of childhood hydrocephalus Rekate and Blitz propose a dual circuit model of hydrocephalus, in which a blockage to CSF flow could be anywhere within either the CSF circuit or the venous outflow of the vascular circuit [13]. Similarly, it can be shown that an increase in fluid flow through either the CSF circuit (through an increase in CSF production) or vascular circuit (from an increase in blood flow) would also elevate CSF pressure. The current study seeks to investigate the incidence of venous stenosis and an elevation in blood flow in childhood hydrocephalus. Given a pressure rise of 6–8 mmHg is required for patients to be symptomatic, what degree of venous outflow stenosis would this equate to? Mathematical modelling of a 7.5 mm diameter cerebral vessel suggests the pressure drop across a stenosis rapidly increases from approximately 2 mmHg at a 50% stenosis, to 5 mmHg at a 60% stenosis, to 10 mmHg at a 65% stenosis and up to 20 mmHg at a 70% area stenosis [16]. From this, we can see that a 65% stenosis or greater is required for the stenosis to be the major cause of an increase in ICP by raising the pressure by 10 mmHg in symptomatic hydrocephalus. Thus, a > 65% stenosis has been designated as significant in this paper. Between 50 and 65% a stenosis may be contributing to the pressure rise, but may not be the major factor, i.e. a non-significant stenosis. In this cohort 31/55 or 56% of patients had a significant stenosis which could account for the majority of the symptoms, whilst 6/55 or 11% had non-significant stenoses. There was one significant stenosis in the superior sagittal sinus, 4 in the transverse sinuses, 4 in the proximal sigmoid sinuses, 17 in the distal sigmoid sinuses and 5 in the jugular bulbs (see Fig. 2). In the latter, the jugular bulb area was less than the adjacent distal sigmoid sinus and was measured instead (see Fig. 2d). With regards to hydrocephalus type, 4/9 patients with external hydrocephalus had significant stenoses, 8/10 patients with obstructed hydrocephalus had a significant stenosis and 19/36 patients with communicating hydrocephalus had a significant stenosis. The findings in external hydrocephalus are similar to a previous study, where 3/6 children had venous stenosis [12]. The findings in obstructed hydrocephalus also mirror previous findings [11, 15] i.e. that a significant outflow stenosis rather than a presumed increase in CSF outflow resistance secondary to aqueduct occlusion predominates.

If an elevation in venous pressure causes both IIH and hydrocephalus, what is the difference? In a large study of 145 pediatric IIH cases, 52% showed dominant-side venous obstruction (with 68% of these showing collateral circulation), compared with 4% of controls showing a dominant-side obstruction [24]. These findings are almost identical to the current study. The age of onset [8] (as already discussed) may be a factor by allowing enlargement of the compliant skull to permit CSF accumulation. Additionally, in the current cohort, the average sagittal sinus was reduced in size by 26% in area. In a recent study in an older hydrocephalus cohort, of mean age 44 years old, there was an average 38% stenosis of the sagittal sinus by area, which generated an increase in the pressure gradient between the superficial and deep venous territories of 1.2 mmHg. This sagittal sinus narrowing was absent in IIH [25]. Thus, a moderate sagittal sinus narrowing appears to correlate with ventricular enlargement in both adults and children. It could be hypothesized that an elevation in venous pressure should reduce the size of the ventricles rather than increase them, however, in adults with IIH the ventricles are unchanged in size by the venous pressure [26]. This suggests that previously enlarged ventricles may not reduce back to the normal range despite an ongoing venous pressure rise.

### Hyperemia

Hyperemia is defined as an increase in the amount of blood in a part, organ or tissue as a result of dilatation of the supplying arteries [27]. In this study the arterial supply to the brain is directly measured at the skull base. In a large study, using an identical MRI flow quantification technique to the current study, the total cerebral blood flow increased from 7 months to 6 years and declined thereafter, averaging 1100 ± 258 mls/min [28]. These findings are very similar to the current study. By chance, it is expected that in a normal distribution, 2.5% of the population will have a blood flow 2 standard deviations above the mean and 0.3% will have a flow three standard deviations above the mean. The current findings of 13% and 4% respectively (see Table 2), are far above those expected by chance alone. Only two of the 7 patients from this cohort with elevated blood flow were found to have either a significant or non-significant venous outflow stenosis. This suggests that hyperemia and venous stenosis are probably not linked but epiphenomena both of which may increase venous pressure and ICP. There was no evidence of AVM, shunting or fistula on the MRVs of these patients indicating the increased inflow would have passed through the brain to increase both CBF and venous flow. The 7 patients with hyperemia have a blood flow increased by between 1.4 and 2.5 times normal compared to age matched controls. Shawcross et al. using a cerebral vasodilator, found a 17% increase in cerebral blood flow (from 69 ml/100 g/min to 81 ml/100 g min) led to a 33% increase in intracranial pressure (from 15 mmHg to 20 mmHg) [29]. Using age matched brain weights from the literature [30], the 7 patients should have a mean CBF of 65 ml/100 g/min if normal but given the 1.4–2.5 times increase in TCBF, they probably average 110 ml/100 g/min. The blood flow in these children would appear to be more than enough to elevate the ICP above 20 mmHg, rendering them symptomatic, however, 6/7 of the hyperemic patients were asymptomatic.

### CSF outflow resistance

In this study, 18/22 or 82% of the patients with active hydrocephalus had a stenosis large enough to be significant and thus symptomatic (see Table 2). This would tend to indicate that the remainder, i.e. 18% of the active hydrocephalus patients may potentially have a significantly elevated R_out_ as the cause of their symptoms. At 7 months of age, the CSF formation rate averages 0.1 ml/min [31]. It must be acknowledged that the CSF formation rate is difficult to measure in humans and the very low net rate noted may incorporate some CSF absorption through the fontanelle at very low pressure, however the net rate is likely to be very low in infants. The normal R_out_ is 2.9 mmHg/ml/min in infants [32]. Using Davson’s equation, this means that in infants, the CSF outflow pressure only makes up 0.3 mmHg of the total ICP of 3.7 mmHg. In order to increase the ICP by 7 mmHg in infants, the R_out_ would need to be 73 mmHg/ml/min. This level would only be obtained if the outflow were totally blocked by, for example acute haemorrhage or meningitis. The 4 infants with active hydrocephalus but without stenoses in the current cohort may fulfil these criteria. Indeed, in a study of 6 infants with active hydrocephalus, the R_out_ only averaged 7.2 mmHg/ml/min [21] suggesting the R_out_ was not high enough in the majority of infants to elevate the ICP and another cause for their active disease must be operating. In older children, the CSF formation rate averages 0.4 ml/min [33]. In these older children, the normal R_out_ is between 5.5 and 10 mmHg/ml/min [5]. Thus, the CSF outflow pressure makes up a maximum of 4 mmHg of the normal ICP of 14.6 mmHg. In 20 children with hydrocephalus between the ages of 3–15 years, the R_out_ ranged from 12 to 30 mmHg/ml/min [34]. In order for a R_out_ to cause a 7 mmHg increase in ICP alone, the value would need to be above 28 mmHg/ml/min. In 6 children with active hydrocephalus, the R_out_ was between 9.5 and 43 [5] and only one child in this cohort, would have had a R_out_ high enough to elevate the ICP by greater than 7 mmHg. It has been shown that an elevated venous pressure in adults with IIH does not elevate the CSF outflow resistance [35] meaning they are separate variables. Thus, we can deduce that chronic active hydrocephalus in children, caused by an isolated elevation in R_out_ on its own is relatively rare.

### Prognostic value

In obstructed and communicating hydrocephalus, a significant stenosis of greater than 65% in the venous outflow appears to be a marker of active hydrocephalus and the lack of a significant stenosis a marker of compensated hydrocephalus. The positive predictive value of a significant stenosis for active hydrocephalus is 67% and the predictive value of the lack of a stenosis for compensated hydrocephalus is 95%. The outcome of external hydrocephalus was almost universally excellent and thus a stenosis or lack thereof had no predictive value. In this cohort, 12/55 or 22% went on to undergo treatment, with 6 having an endoscopic third ventriculostomy (ETV) and 6 a ventriculoperitoneal shunt (VPS). A significant venous outflow stenosis appeared to be a risk factor for the failure of conservative treatment, with 10/12 or 83% of treated children having a stenosis greater than 65%. The failure of treatment to completely abolish the stenoses appeared to be a marker of ongoing symptoms. In both of the children where surgical treatment failed to completely resolve the stenoses their symptoms remained. In contrary, an elevation in TCBF appeared to be a good prognostic indicator, with none of these 7 patients requiring surgery. The reason for this later finding may relate to a possible reduction in the blood flow, back to the normal range, occurring with development.

The current hydrocephalus paradigm implies that the vast majority of children with hydrocephalus have an elevation in R_out_ as its sole cause. Thus, treatments are designed to improve the R_out_ by either unblocking the third ventricle (ETV) or adding an additional outflow path (VPS). However, this treatment is not without morbidity. The incidence of ETV failure at 2 years is about 35% [36]. Shunt infection is in the order of 5% per insertion [37] and shunt failure from obstruction occurs in 40% of children in the first 2 years after placement [38]. The continuation of a venous stenosis following treatment may allow prognostication regarding some of these treatment failures but further work is required.

Given descriptions of high grade stenoses in childhood hydrocephalus appear in the literature from the early 1960s [9, 10], one may ask “why has there been so little interest in this finding?” The current paper being retrospective is unable to answer the question of causality directly. However, in a paper by Sainte-Rose et al. the possibility of causation was tested [7]. There were two basic theses underlying this paper i.e. (1) that the stenoses found were due to venous collapse caused by the elevation in ICP associated with the hydrocephalus and therefore not causative and of no therapeutic interest and (2) the stenoses were fixed and not caused by the elevated ICP and therefore potentially of therapeutic interest. The findings in achondroplasia were that the stenoses were fixed and probably causative with regards to the hydrocephalus. It is of note that in two of the current cohort, the stenoses failed to completely resolve with treatment and the patients remained symptomatic. However, the further findings by Sainte-Rose et al. were that the majority of the intracranial stenoses were due to venous collapse secondary to the ICP and it was concluded the stenoses should not be treated. This has remained the conventional wisdom since. However, it has subsequently become apparent that thesis one and thesis two are not mutually exclusive. In idiopathic intracranial hypertension in adults, venous collapse has been found to be both caused by the elevated ICP and to cause the elevated ICP through an unstable positive feedback loop [39]. Stenting these stenoses breaks the feedback loop and appears to cure the vast majority of adults with IIH who have a focal stenosis with a pressure gradient above 8 mmHg [40]. Both IIH in adults and hydrocephalus in children are analogous and have apparently identical reversible stenoses secondary to venous collapse [7, 40]. In those with fixed stenoses from achondroplasia either a venous bypass graft [7] or jugular bulb decompression has led to cure [41]. It is currently unknown if stenting venous stenoses secondary to collapse in childhood hydrocephalus could convert active to compensated hydrocephalus or even induce a cure in some, thus we believe further work is warranted in this area.

## Conclusions

The elevation in ICP in symptomatic hydrocephalus is multifactorial. Both high grade venous stenoses and cerebral hyperemia are common in childhood hydrocephalus. High grade stenosis is noted to be a risk factor for failure of conservative management. Hyperemia is a good prognostic indicator.

## Data Availability

All data generated or analysed during this study are included in this published article.

## Abbreviations

CVP: Central venous pressure
D_e_: Effective diameter
ETV: Endoscopic third ventriculostomy
FLAIR: Fluid attenuated inversion recovery
FR_csf_: CSF formation rate
Hd: Hydraulic diameter
IIH: Idiopathic intracranial hypertension
mmHg: Millimeters of mercury
MRV: Magnetic resonance venography
R_out_: CSF outflow resistance
SSS_p_: Superior sagittal sinus pressure
TCBF: Total cerebral blood flow
VPS: Ventriculoperitoneal shunt
